# Help-seeking for mental health and substance use problems among people in contact with the criminal justice system: a mixed-methods social network analysis

**DOI:** 10.1186/s40352-026-00431-2

**Published:** 2026-06-26

**Authors:** Catriona Connell, David Griffiths, Jessica Greenhalgh, Morgan Torrance

**Affiliations:** University of Stirling, Stirling, United Kingdom

## Abstract

**Background:**

Mental health (MH) and substance use (SU) problems are prevalent among people in contact with the criminal justice system, many of whom serve all (or part) of their sentence in the community. Barriers to help-seeking in this population contribute to insufficient support, and later serious adverse outcomes for individuals and society. We aimed to understand variation in the relational context surrounding people in contact with the criminal justice system, and how these differences influence help-seeking for MH or SU problems.

**Methods:**

We interviewed 50 people in contact with the criminal justice system in a mixed-methods social network analysis study. We collected data about participants’ social networks, help-seeking experiences, perceived societal stigma and perceived usefulness of MH and SU support. We developed a network typology by synthesising these data, and explored relationships between network types and help-seeking for MH or SU problems.

**Results:**

We demonstrated the feasibility of our exploratory approach to developing a typology of social networks among people in contact with the criminal justice system in the community. Five network types were differentiated by structure, composition, network members’ characteristics, and culture. These could be applied to explore influences on a range of behaviours. Knowledge and attitudes towards MH, SU, and help-seeking across the network and within the wider community were important influences on individual help-seeking behaviour.

**Conclusions:**

Interventions to encourage help-seeking, and other behaviours, among people in contact with the criminal justice system should consider network type and relational context. Introducing diverse contacts who can provide alternative perspectives may be useful for changing network culture towards different behaviours and facilitating individual change. Tailored messaging and delivery methods are required to increase awareness of MH and SU problems, and of when, where and how to seek help across the networks of people in contact with the criminal justice system. Further research could test our exploratory approach in larger samples and across contexts, examine the impact of network change over time, and rigorously develop and test relationally informed and context-responsive interventions.

**Supplementary Information:**

The online version contains supplementary material available at https://doi.org/10.1186/s40352-026-00431-2.

## Background

Many people convicted of a criminal offence serve all or part of their sentence in the community. In several European countries, the population serving a sentence in the community is larger than that in prison with approximately 2.4 million people across Europe under probation supervision at any time (Aebi et al., 2025). Community sentences take different forms internationally, and based on the offence and personal characteristics of the person convicted, but include supervision following release from prison (e.g., probation/parole), and community sanctions and measures without time in custody (e.g., conditions on movement and behaviour, treatment orders, unpaid work/‘community service’) (Aebi et al., 2025).

Prevalence of mental health (MH) and substance use (SU) problems is high among people serving sentences in the community, with substantial unmet need (Sirdifield et al., 2024; Sirdifield 2012). Access to adequate support for MH and SU problems is essential, given the elevated rates of contact with emergency services for MH/SU-related concerns (Connell et al. 2026; Frank et al. 2013, 2014) and deaths attributed to SU and suicide (Borschmann, Keen and Spittal 2024; Slade et al. 2024). Further, community supervision has been implicated in worsening MH and SU, (Phelps et al. 2022; Tuschick et al. 2024) emphasising the ethical responsibility of states and services to ensure adequate care is available and accessible for this population. People in contact with the criminal justice system in the community must typically access ‘mainstream’ MH or SU services, which may not be optimally structured or resourced to work effectively with people living with the challenging life circumstances often experienced by this population (Bramley et al. 2015).

Help-seeking is a necessary step towards accessing healthcare (Levesque et al. 2013; Pescosolido 1991). Help-seeking involves taking steps to gain assistance, for example calling a service provider or crisis line, or approaching a trusted other. This may result in receipt of support but does not guarantee it (e.g., screened out of a service, other person is unsupportive). Help-seeking thus differs from receiving help, and from healthcare utilisation which indicates the person has received (at least some) help. 

Help-seeking can be conceptualised as a planned human behaviour, based on a conscious decision to act, that results from interacting individual attitudes, perceptions of norms within one’s social network and community, and perceived ability to act (Ajzen 1991). Existing literature demonstrates an association between attitudes, norms, ability and MH help-seeking intentions (Adams et al. 2022). Few studies examine SU help-seeking or measure help-seeking *behaviour,* with measurement of behaviour typically being evidence of its presence or absence rather than rating scales. Further, there is limited evidence supporting existing models in socially disadvantaged populations. 

Attitudes, perceptions of norms, and perceptions of abilities are socially mediated, that is, they are influenced by and through interaction with people in social networks embedded in local contexts and communities (Berkman et al., 2020; Marsden and Friedkin 1993). Social network approaches are increasingly applied to understand variation in health outcomes (Pomare et al. 2022), particularly personal network methods which examine how an individual’s social connections impact their lives. Ego-centric networks (Crossley et al., 2015) use contextual information about the structure and composition of people’s networks to understand the relational influence on their behaviours. This can extend to creating network typologies and examining their effects (Vacca 2020). For example, a typology of care home residents networks has been developed using structural features (whether all named people formed a single component, whether all were connected and shared information), compositional features (whether a care worker was in the network), and qualitative data to help understand residents’ differing needs (Ferguson 2021).

The social and relationship challenges experienced by people in contact with the criminal justice system indicate that there are likely to be distinct factors shaping their social networks that may impact help-seeking. The nature of an offence may cause relationship tension, relationship loss, self-isolation, and harassment (Tewksbury and Lees 2006), all of which may deter help-seeking. A period removed from the community can contribute to relationship fragmentation and replacement (Volker et al. 2016). When criminal justice system contact intersects with MH and SU problems, networks can be further strained or enhanced to hinder or facilitate help seeking. For example, isolating oneself due to shame or anticipating stigma, gaining members who support substance use or recovery, or the substitution of friends or family with service providers. People may avoid friends due to the potential for information about offending or health to spread in their network and community. The size of networks and roles of the people within them may change, which can reshape pathways to seeking help, for example fewer colleagues due to employment loss, and more service provider contact. 

Peoples’ social networks sit in distinct national and local contexts. Scotland has the highest rates of community supervision, and among the highest rates of imprisonment in Western Europe (Aebi et al., 2025). Recent efforts have been made to reduce stigma towards MH and SU nationally (Scottish Government 2021; SAMH 2025), the latter accelerated by the rise in the drug related death rate, that dwarf rates in comparable nations (National Records of Scotland 2025). Drug-related deaths disproportionality occur among people with a history of imprisonment, and in communities impacted by socioeconomic hardship (National Records of Scotland 2025), where many people in contact with the criminal justice system reside (Public Health Scotland 2026). In Scotland, MH and SU treatment is universal, free at the point of use, and paid for through general taxation. However, capacity constraints limit services' abilities to meet the level of population need and complexity, particularly for people experiencing mental health distress and where this experienced alongside concurrent SU needs (Scotland 2023). MH and SU services are organised regionally, and often (though not always) accessed via referral from a general practitioner (primary care physician). MH services are predominantly provided by local health boards, and SU services by both health boards and local authorities (also called local councils or local government; organised independently from national government to provide services to their locality) working in partnership. Health board and local authority services are complemented by voluntary sector providers, though provision varies geographically. Local authority justice social work services support and supervise people in the community in contact with the criminal justice system. Social workers can provide a link to other services but not privileged access, even where there are mandatory treatment requirements as part of someone’s community sentence. 

Whilst studies increasingly explore networks typologies in relation to MH (Litwin et al. 2020; Usmanov et al. 2022) and SU, (DiGuiseppi et al. 2020) few contemporary studies examine the personal networks of people in contact with the criminal justice system in the community (Goodson-Miller 2022) and there is limited knowledge about how social networks in context influence help-seeking for MH and SU problems in populations experiencing marginalisation (Connell et al. 2025). Scotland, with its high criminal justice contact rates and levels of harm attributable to suicide and SU (National Records of Scotland 2023, 2024, 2025), provides a valuable context in which to develop better understanding the social networks of people in contact with the criminal justice system and their relationship to help-seeking. This can inform more effective population-, network-, and individual-level interventions to increase help-seeking, and other behaviour. 

### Aim

We aimed to develop a network typology to better understand how social networks influence help-seeking for MH and SU problems among people in contact with the criminal justice system living in the community in Scotland, and to inform policy and practice interventions to maximise successful MH and SU help-seeking.

## Methods

### Study design

We used mixed-methods social network analysis with a convergent parallel design. Data were collected at interview with people in contact with the criminal justice system, living in the community in Scotland. Study protocols are available (Connell et al., 2024).

### Ethical approval

The research was conducted in accordance with the Declaration of Helsinki principles. Ethical approval was obtained by the General University Ethics Panel, University of Stirling (13,947). Additional ethical approvals were obtained from Community Justice Scotland and The Salvation Army, who supported recruitment.

### Sample

We included adults (18 + years) who had been in contact with the criminal justice system (courts, prison or probation/community justice) within the past six months, who lived in one of two local authority areas in Scotland, who could be interviewed safely in a community setting, and who could give audio-recorded verbal informed consent. We focused on criminal justice system contact within the past six months to ensure experiences were relevant to contemporary community dynamics and service provision. To identify contextually distinct influences, we selected recruitment sites that differed in levels of deprivation, health inequalities, culture, and urbanicity. Local Authority 1 (LA1) is characterised as relatively affluent, with a large rural landmass and predominance of the farming and fishing industries. Local Authority 2 (LA2) is characterised as highly deprived, small with a central town, and heavily impacted by de-industrialisation.

### Recruitment

We worked with justice social work teams and voluntary sector organisations who identified people receiving their services who would be willing to meet a researcher. Whilst we intended to use both providers in both locations, LA1 recruitment was predominantly through justice social work services, whilst in LA2 recruitment was through a voluntary sector network. When a potential participant agreed to meet a researcher, a researcher attended a community location that the person was familiar with to provide study details, answer questions, and, if appropriate, take consent to participate.

### Data collection

Through semi-structured interviews facilitated by Network Canvas software (Hogan, Melville and Phillips 2016), we collected participant demographics and three types of data. Social network data captured the participant’s perspective of network members’ demographic characteristics, attitudes towards help-seeking, and experiences of MH problems, SU problems, and contact with the criminal justice system. Qualitative data included exploration of participants’ experiences of MH and SU problems, help-seeking, services in their locality, and any topics they considered important. Quantitative data included: whether or not the participant was currently seeking or receiving help for MH or SU; perceptions of the helpfulness of different sources of support for MH and SU (professional, peer and friends/family) rated on a five-point Likert scale; and perceptions of societal stigma towards MH and SU, measured using the 12-item Perceived Societal-Level Devaluation-Discrimination Scale (Link et al. 2015). Wording in the latter was adapted for accessibility. All questions and response coding are available in our protocols (Connell et al., 2024). Interviews were transcribed verbatim and pseudo-anonymised by removing all names of people and locations. Quantitative and social network data were exported from Network Canvas (Hogan, Melville and Phillips 2016) and anonymised.

### Data analysis

We aimed to develop a network typology consisting of a plausible number of network types that that could be meaningfully distinguished from one another, both qualitatively and statistically, and which would be useful in practice. We separately analysed the qualitative, quantitative, and social network data, before triangulating and synthesising results and findings to develop a typology. Qualitative data were analysed with NVivo14, (Lumivero 2023) and quantitative and network analysis were conducted in R (R Core Team 2021) using the tidyverse, egor, igraph, and ggnetwork packages.

To determine the optimal method and number of network types to describe the data, we utilised an exploratory approach combining manual and algorithmic clustering methods, as our small sample limited the applicability of established clustering approaches (Vacca 2020). First, we visualised all networks, then manually sorted them into groups based on visual appraisal of the structural features (size, number of components, density), types of relationships (e.g., friends, family, service providers), and the presence of network members with contact with the criminal justice system (current or past, vs no or don’t know). Relationship types have been demonstrated to be important in help-seeking among people experiencing MH problems (Fulginiti et al. 2016; Perry and Pescosolido 2015), whilst we hypothesised that there may be shared cultures between people with experiences of contact with the criminal justice system. Following this we explored results from clustering algorithms (k-means and k-medioid) using structural features as variables including: size of network; number of edges (connections between network members); number of components (communities of network members who are connected to each other); edge density (the proportion of all possible connections which were made); transitivity (a measure of connectivity of groups of three people); and average path length (average distance between two people in the network). We removed participants whose networks consisted of two or fewer people because this affected calculation based on structural features and prevented successful clustering. We applied the elbow (Thorndike 1953) and silhouette (Rousseeuw 1987) methods, and appraised the within cluster sum of squares and silhouette widths respectively.

We considered the optimal number of clusters suggested by each method and the fit to the data indicated by the silhouette scores. Silhouette scores range from −1 to + 1, and whilst there are no specified parameters, we judged a score of < 0.5 to be indicative of relatively poor fit. Using the optimal number(s) for k determined by each method, we manually appraised visualisations of the networks within algorithmically generated clusters, using colour coding to represent different relationships (e.g. family, friend) and shapes to represent network members’ experiences of criminal justice system contact. We selected the clustering results that made substantive ‘real world’ (based on human appraisal of visualised netowrks) as well as best statistical sense. Each cluster represented one network type within the typology. We sought to obtain a clinically useful number of network types within the typology that that could be meaningfully interpreted and qualitatively (rather than solely statistically) distinguished from one another by human appraisal. We considered two or fewer network types to be too low to reflect the variability in peoples’ lives, and greater than six to be too large for to be practically useful.

To further test the validity of our clustering results, we examined the qualitative and quantitative data, comparing and contrasting between participants within and across network types. To facilitate this, we applied framework analysis (Gale et al. 2013) to understand the individual, network, and contextual factors that influence MH and SU help-seeking, informed by theories of human behaviour and help-seeking (Levesque et al. 2013; Pescosolido 1991; Ajzen 1991). In this paper, we examined network factors in depth, considering how this may influence network structure and help-seeking among participants. The full qualitative framework is included in our Supplementary File, and results are reported elsewhere. We have ‘cleaned’ verbatim quotations (translating words from local dialect/language/slang, presenting in English spelling) for the international readership.

We calculated descriptive statistics for the sample and compared these across network types for informative purposes, recognising that number of participants within each network type was too small for robust statistical comparisons. Quantitative data were nonetheless considered useful to triangulate with the qualitative findings.

We examined differences between network types in the proportion of participants either receiving or seeking help. We also examined variables known to contribute to help-seeking: stigma and perceived helpfulness of different sources of support. These variables, in part, give insight into to underlying attitudes and perceived social norms that may motivate help-seeking and impact accessing the right assistance.

### Patient and public/lived experience involvement

A Lived Experience Advisory Panel, consisting of people with experience of contact with the criminal justice system and help-seeking for MH and SU, met regularly with the research team to advise on study design, interpreting results, naming the network types, and preparing dissemination materials. The Lived Experience Advisory Panel provided guidance on adapting the scoring of questions about the helpfulness of support, and amending the wording of the Perceived Societal-Level Discrimination and Devaluation Scale to make the language acceptable in a Scottish context, and more comprehensible to the participant group. We held discussions with local service providers, and their representatives joined a study advisory group.

## Results

We interviewed 50 justice-involved people, with interviews lasting between 28 and 301 min (mean 90 min). Two participants with networks of 1–2 people were excluded from analysis. The total sample was 48; twenty-two participants from LA1, and 26 from LA2.

Participants had a mean age of 38 years and were mostly male (71%) and white Scottish (88%), reflecting the demographic characteristics of the Scottish population in contact with the criminal justice system. Sixty-seven percent had a community sentence (e.g., unpaid work). Sample characteristics are summarised in Table 1.

**Table 1:** Sample characteristics

Characteristic	*N* = 48^1^
Age	38 (10)
Gender	
Male	34 (71%)
Female	14 (29%)
Ethnicity	
White Scottish	42 (88%)
Other	6 (13%)
Local authority	
LA1	22 (46%)
LA2	26 (54%)
Area deprivation level	
20% most deprived	36 (75%)
Other	12 (25%)
Type of justice involvement	
Community disposal	29 (67%)
Other	14 (33%)
Has current MH/SU condition	44 (92%)
Has MH/SU support	37 (77%)
Currently help-seeking	28 (58%)
Previous failed help-seeking attempt	38 (79%)
Ever used NHS/LA services	37 (77%)
Ever used peer support	23 (48%)

^1^Mean (standard deviation); number (%)

*LA *local authority, *MH *mental health, *NHS *national health service, *SU *substance use

Most participants were experiencing MH or SU problems (92%), accessing MH or SU help (77%), and seeking (additional) help (58%). MH and SU services provided by the health service or local authorities were more commonly experienced (77%) than peer support (48%). Most participants had experience of seeking but not receiving help (79%).

### Typologies

**Fig. 1:**
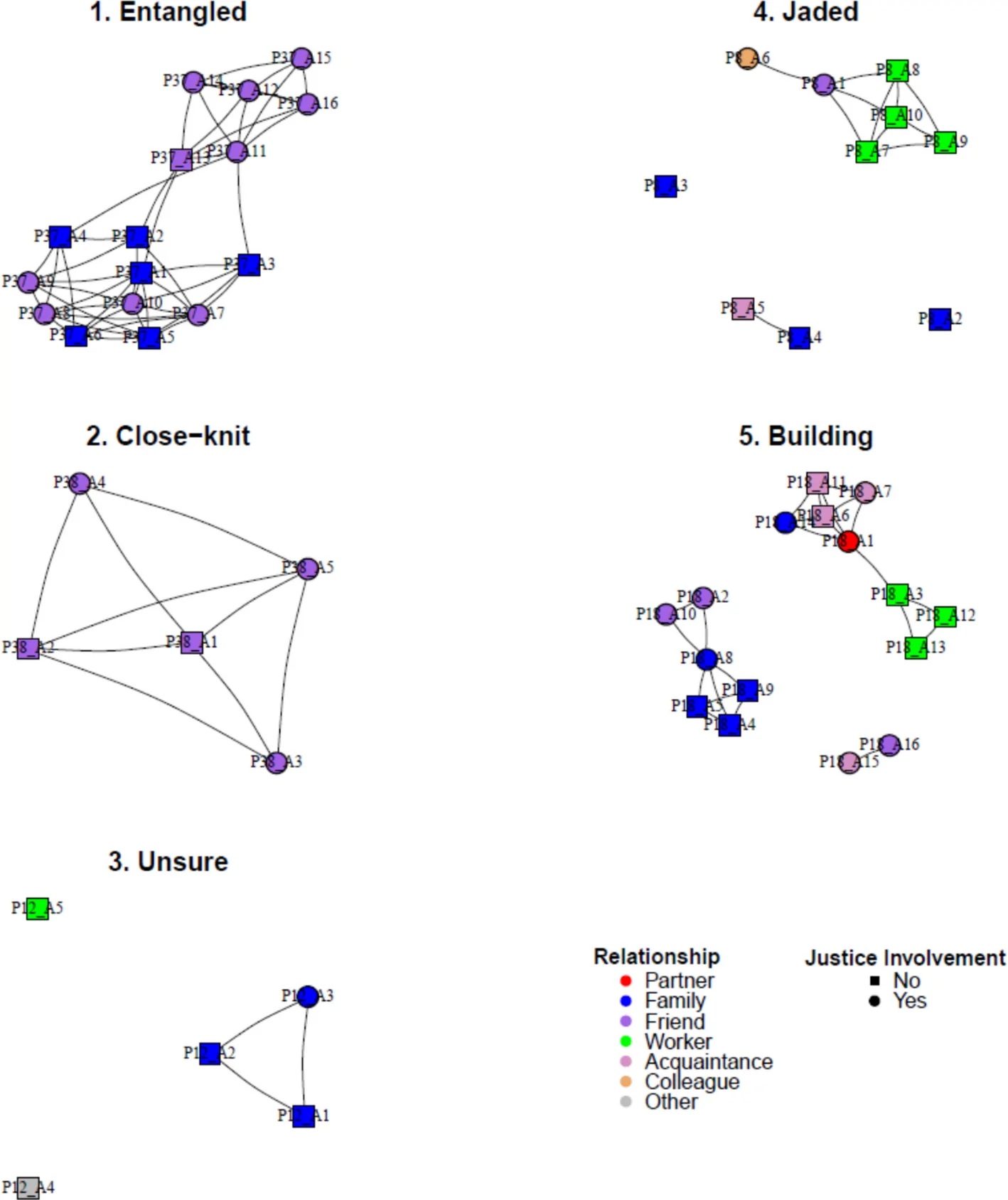
Network typology with representative network for each network type

Our mixed-methods synthesis produced five network types: ‘Entangled’, ‘Close-Knit’, ‘Unsure’, ‘Jaded’ and ‘Building’. Figure 1 shows the network typology, illustrated by a representative network from each network type. Table 2 shows the distribution of the network types across the sample, with a description of each network type.

**Table 2:** Distribution of network types with description

Type	Frequency	Description
Entangled	10	Larger networks with closely connected members. Lowest levels of supportiveness for help-seeking and fewer service providers. Friends were important and were often longstanding relationships. People could be fearful of losing their identity within the network which deterred help-seeking, but with the larger proportion of friends they had the opportunity to see a friend role model positive change
Close-knit	16	Small networks with closely connected members. Fairly high levels of help-seeking. Often made up of a single relationship type which could limit knowledge available to someone and the attitudes they were exposed to
Unsure	8	Small network with a core component and single peripheral contacts. Peripheral contacts could be crucial for introducing new perspectives about help-seeking. People were often uncertain about help-seeking and the motives of others who may offer to help
Jaded	6	Mid-sized networks made up of a main component and other small components. People were deterred from help-seeking by negative experiences of primary care and specialist services. They had relatively high proportions of service providers in their networks, often social work/social support. People described securing support by committing crime, enabling them to sustain a publicly antagonistic position towards services while privately receiving help. Slow trust-building through practical assistance before approaching MH or SU was important
Building	8	Larger networks made up of several mid-sized components. Higher levels of supportiveness for help-seeking, and high levels of shared MH, SU and experiences of contact with the criminal justice system. Friends were important but were more likely to be newer and selected friends who shared desires to improve their health and move away from criminal activities

In determining optimal cluster numbers, our visual appraisal indicated five clusters, and the elbow method suggested four or five for both k-means and k-medioid clustering. The silhouette method suggested two, seven or eight for k-means, and three or five for k-medioid clusters. However, silhouette scores, even when optimised, still suggested poor fit (below width 0.5). Visually reviewing the outputs of statistical clustering showed that five clusters could be meaningfully distinguished by human appraisal in the k-means clustering results, consistent with our initial visual appraisal that determined five clusters.

**Fig. 2:**
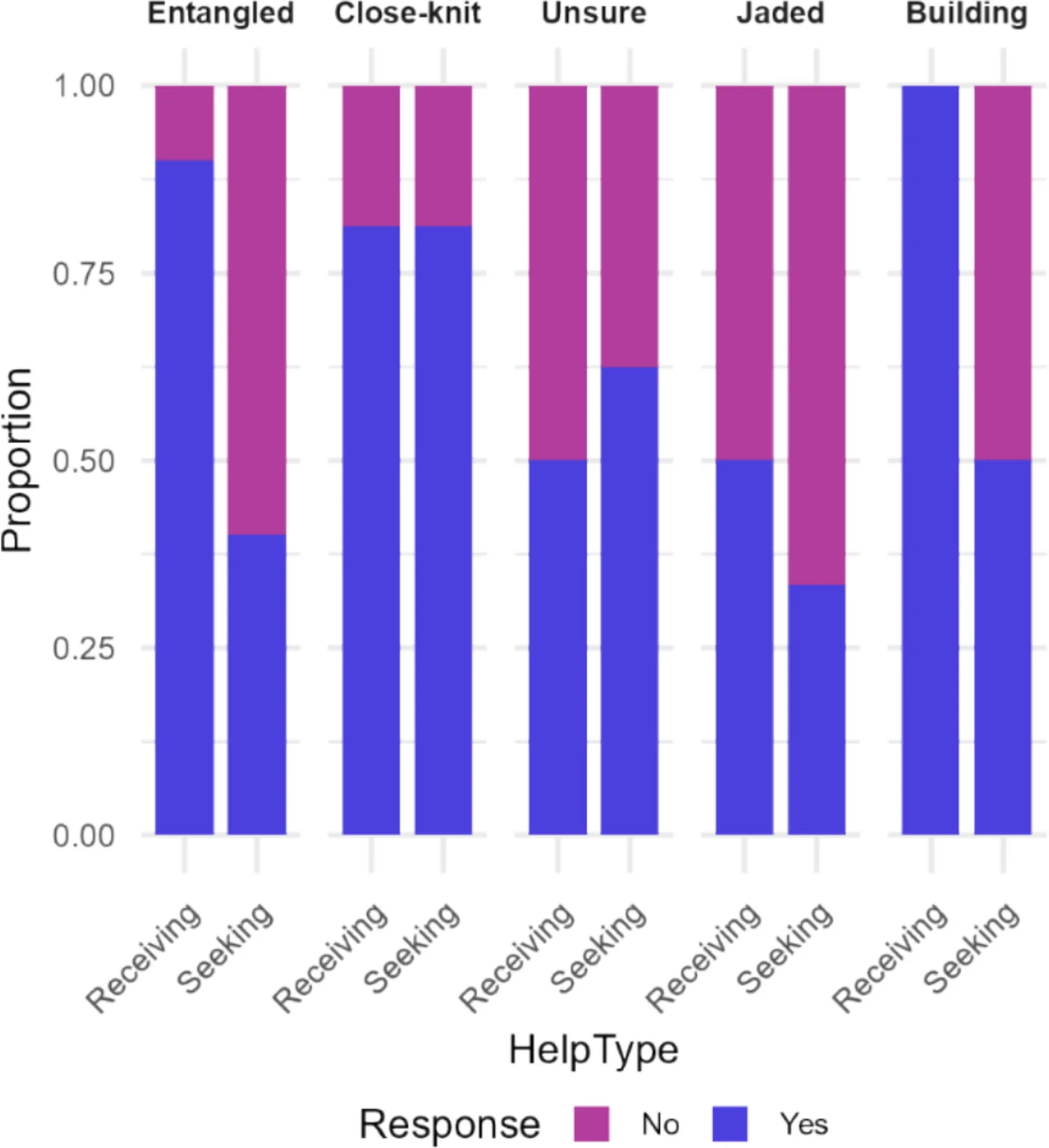
Proportion of participants receiving and seeking help by network type

Quantitative between-network differences should be considered descriptive (due to the small number of participants in each cluster preventing statistically robust comparison). However, complemented by the in-depth qualitative findings, they provide valuable insights. Variation in the levels of help-seeking and access to help was observed between the network types, as shown in Fig. 2. Supplementary File tables A and B show the distribution of individual variables and network characteristics across network types.

On average the sample perceived higher levels of societal SU stigma (mean [M] = 1.86, standard deviation [SD] = 0.36) compared to MH stigma (M = 1.49, SD = 0.45), a pattern that was replicated across network types. For both MH and SU, on average family and friends were perceived to be least helpful (MH M = 2.25, SD = 1.41; SU M = 2.21, SD = 1.44) compared to professionals (MH M = 2.60, SD = 1.33; SU M = 2.9, SD = 1.10) or peer support (MH M = 2.79, SD = 1.09, SU M = 2.79, SD = 1.18). A score of 2 on an individual basis would indicate a rating of ‘neither helpful nor unhelpful’ and 3 ‘helpful’. This pattern was also replicated across network types, but divergence in scores were evident between participants, indicating a need to treat averages with caution. Supplementary File Table C shows scores for perceived stigma and perceived helpfulness of different types of support overall and across network types.

Examining qualitative data revealed similarities across network types that may be common experiences for people in contact with the criminal justice system in the community. These are presented first followed by presentation of the network types.

#### Network commonalities

Participants described how their networks lacked knowledge about MH and SU problems and how best to support these needs. Consequently, network members could not identify problems early and support help-seeking effectively.

"I grew up in an addictive household and all…I just thought that was kind of normal to just mask your feelings and how you felt and use drugs and drink and I just thought that’s the way it was." [P46]

"because I don’t know how to go about it because I will be asking them for help and they can’t help me...” [P13]

Most participants deliberately shaped their networks in ways that supported or undermined help-seeking, by ‘cutting people off’ or imposing a self-isolation. Cutting-off was mostly seen in relation to substance use, where people ceased contact with others who used substances or who may sabotage their efforts to leave substances behind. However, cutting-off could be challenging in areas where substance use was prevalent.

*“it’s just because of my past. I have to do that to keep myself clean, to keep myself- and again, it comes back to the problem with drugs [in the] area, it’s basically everybody does it.*" [P2]

A few participants had cut off, or been cut off, by people who disapproved of their SU. Consequently, they were left with fewer options and tended to associate with people who wouldn’t confront them.

"Because I know they would condone it, d’you know what I mean." [P42]

To a lesser extent this was seen in relation to mental health, where people avoided others who may cause or exacerbate mental distress.

*You kind of reinforce each other’s shitty period." [P5].*

The resulting isolation and loneliness were often seen as the necessary price for protecting themselves, but not without the associated risks of having limited social support.

*I keep myself to myself to a bad extent, yes. Because I don’t want to be- Like hang about with the wrong people and there’s a higher risk of relapsing" [P6].*

Stigma towards MH and SU was perceived and experienced across network types, with participants aware of others’ views about people with MH or SU problems. Many had experienced and anticipated negative appraisal by others, including from healthcare providers, which deterred help-seeking.

#### Entangled

Entangled networks were larger and had medium-levels of interconnectedness between network members. They were characterised by higher numbers of friends experiencing similar challenges with MH, SU, and contact with the criminal justice system. Entangled networks had a lower proportion of service providers. A higher proportion of network members were unsupportive of help-seeking for MH or SU compared to the other network types. Overall participants with Entangled networks had high levels of contact with the criminal justice system.

Participants with this type of network spoke about friends and their importance for MH and SU help-seeking. They were more likely to talk about long-term friendships. For a few participants, seeing long-term friends make positive changes and having trusting relationships with them was fundamental for their own help-seeking.

*“But, the past two/three years, all my friends I’ve seen take the next step. So, I’ve seen my friends take the next step, so if you can do it, so can I." [P11]*

However, participants could be deterred from help-seeking for fear of a negative impact on the identity they wanted to project to their network, and the status they felt they had through contact with the criminal justice system.

*"half the time I’m thinking inside in my actual head and my body; how do I ask this person, what will I say? I don’t want to ask them in case I look an idiot. So, I just say fuck all." [P19]*

#### Close-knit

Close-knit networks were smaller and medium-highly interconnected. These networks were typically dominated by one relationship type, usually either family members or service providers. Participants with Close-knit networks had fewer ties to people who faced similar challenges to themselves, but similarly high levels of unsupportive network members as participants with Entangled networks.

There was variation in culture of these small networks. Some participants had Close-knit networks that were highly encouraging of help-seeking, and others had unsupportive cultures. Both could limit the sources of knowledge available to participants.

*"I think it’s just because everybody is just so closed off to an idea of anybody helping you now, do you know what I mean? I think that’s the mindset of addicts" [P14]*

*"I don’t speak to anybody else that’s not in NA [Narcotics Anonymous], so it’s like friends are all right now" [P32]*

Many participants were aware of their small networks which could contribute to hesitance, in case help-seeking damaged their relationships or credibility with peers. This group identified a concern about upsetting network members by seeking help.

*“it can worry them do you know what I mean? I think you are better speaking to a professional but I think most family members and stuff would always try and advise you to go and seek help but they aren’t armed with the facts" [P46]*

Overall participants with Close-knit networks were wary of others, finding social contact stressful. The fear of information about them spreading made them reluctant to discuss their difficulties.

*"Talking behind your back. They wouldn’t say it to your face but they actually behind you everywhere, know what I mean.” [P26]*

Often these participants perceived higher levels of societal MH stigma and applied this harshly to themselves. Many described how negative or invalidating experiences with health professionals/peer support contributed to a sense that no-one could or would understand and help them, reinforcing negative self-perceptions**,** anticipation of stigma, and deterring help-seeking.

*"They just said I was a bit sad. And it made me feel like, oh, so I’ve overreacted. I’ve just tried to kill myself because I’m a bit sad. And it just made me feel weak.” [P1]*

#### Unsure

Unsure networks were made up of a main component (group of connected people) with another small component/single contact. Only half of participants with this network type were currently receiving support. Participants with Unsure networks were relatively young on average (mean age 35 years) and had the lowest levels of criminal justice system contact, potentially indicating a greater openness to help-seeking.

Unsure networks had lower proportions of people with criminal justice, MH and SU experience, but high levels of support for help-seeking. They had a high proportion of network members defined as service providers and lower proportions defined as friends.

Peripheral contacts could play an important role. In this example a young man talks about how a contact spoke about his alcohol use, making talking about challenges appear acceptable.

*“So they do talk about it so if I did have an issue with alcohol I would definitely mention it to [men’s group] and someone else in the group would go, oh well I’ve been down that route this is what it did to me this is how I got help." [P3]*

Many participants with Unsure networks described how help-seeking was something they (initially) would not consider as a course of action.

*“other people asked me to go to the doctors about mental health problems and stuff like that. I ignored it because I didn’t want to do it, somebody else telling me what to do." [P12]*

Many were suspicious of the motives of support services, including peer support, having had experience of their trust being broken or growing up in cultures that rejected authoritative organisations.

*"I have learned not to trust the system at all, I don't think that anyone is genuine like, they all have agenda." [P49]*

#### Jaded

The Jaded networks consisted of a few variably sized, disconnected components. Jaded networks were the least common (*n* = 6) and were predominantly seen amongst those residing in the more rural LA1. Participants with Jaded networks were older on average and had less criminal justice system contact compared to participants with Entangled and Close-knit networks.

These participants were least likely to be seeking MH or SU help, despite high identified levels of need. Like participants with Close-knit networks, they described negative experiences with general practice, and MH and SU services, that deterred them from help-seeking. Participants with Jaded networks also described internalised cultural norms not to seek help.

*"from when we were brought up, if you asked for anything, you got a hiding. So, the best way is not to ask.” [P21]*

Participants in Jaded networks had the highest proportion of service providers in their networks, who were often social or criminal justice rather than health workers. This greater presence of professional service providers highlights the limited support available through family, friends and peer networks.

*“I would speak to my social worker or my solicitor" [P28].*

For some participants**,** orchestrating access to social work through criminal activity was the only route they felt they had left following negative healthcare experiences.

*"I’ve recommended it to a few folk, because they weren’t getting help, and they did it and I was right, and they got help because they committed crime” [P8].*

Due to the more sceptical position about services among these participants, a sustained relationship and support built over time was particularly important if someone was to seek help at a time of later difficulty.

#### Building

Building networks varied in size. Like Entangled networks, they tended to have few components, but in contrast these components were more distinct and closely inter-connected. Building networks more common among participants from LA2. Most participants with Building networks had ‘other’ justice involvement (including being on bail or recent sentence completion). Building networks had a high proportion of friends and the highest proportion of network members in contact with the criminal justice system. Friends were important influences on help-seeking within these networks, however unlike in Entangled networks, more attention was given to new friends who shared the participants’ aims to improve health.

*“they’ve all been through it, so, they can see the difference in me and they can see the difference in other people, and the difference in themselves. So, yes, that’s the only people that I really want to be associated with now." [P47]*

Though many participants spoke positively of peer support, some participants were sceptical about or unable to access it, the latter tending to be those in LA1. This was consistent with lower levels of having ever experienced peer support among participants in LA1.

*"sat in a room with a bunch of addicts really doesn’t make sense to me" [P5]*

“*if I’d seen like a poster up or anything like that and they had things like AA meetings or anything like that in [location] or like a mental health group where you can go and meet people or people that’s had past experience I would definitely go" [P18]*

Many network members also had experience with MH and SU, but across the Building networks there were fewer network members who were actively unsupportive of help-seeking for MH and SU. This suggests that, unlike Entangled networks where there were high levels of shared experience and higher unsupportiveness, the culture of Building networks may be more encouraging of help-seeking.

*"In [location], I suppose, asking for help people kind of go, “Oh, she’s actually doing it,” whereas in [location] it’s [SU] just normal” [P38]*

Participants in Building networks perceived lower levels of societal stigma, potentially indicating that these larger, more diverse and supportive networks have a less stigmatising culture. Participants with Building networks did not internalise stigma to the degree seen among those with Close-knit networks but were still cautious and selective about help-seeking.

*“to certain people, yes, or like say that people was like able to see me asking someone else, like if it was to a big like network or something like that and they’d seen that then that would restrict me from going to ask, yes. " [P18]*

## Discussion

This study applied novel mixed-methods approach to develop a network typology with people in contact with the criminal justice system living in the community. We interviewed 50 people in Scotland about their experiences and perceptions of seeking help for mental health (MH) and substance use (SU) in their communities, societal MH and SU stigma, and the characteristics of their social networks. We analysed social network, quantitative, and qualitative data to create a network typology, and explored the relationship between network types and help-seeking.

We demonstrated the feasibility of developing a typology of social networks among people in contact with the criminal justice system in the community. Our small sample, consisting of people with relatively small networks (sometimes one or two people), prevented the application of documented social network clustering methods (Vacca 2020). Consequently, the optimal approach to typology development involved combining qualitative appraisal of the network visualisations and exploratory cluster analysis, before triangulating this with quantitative results and qualitative findings. The network typology was created without reliance on data specific to MH or SU. Thus, whilst we explored how network types related to help-seeking for MH and SU, they could plausibly be used to understand relational influences on wider health or offending behaviour in the population.

Participants with all network types described how their help-seeking was impacted by a lack of knowledge in their network, perceived and actual stigma, and whom they ‘cut’ from their networks. We observed greater perceived societal stigma towards SU compared to MH, and qualitatively showed how help-seeking could be influenced by this (via anticipatory and experienced stigma including from service providers) and self-stigma, particularly in small close-knit networks. In studies using general population samples, MH help-seeking has been associated with personal- and self-stigma, but not perceived societal stigma (Schnyder et al. 2017). Mixed associations have been found between all stigma types and SU help-seeking, though societal stigma was an evident influence and more strongly experienced by those with intersecting stigmatised experiences/characteristics (Hammarlund et al. 2018). Anticipatory and experienced service provider stigma were a barrier in this population, reflecting a recognised international issue. (e.g., Magnan et al. 2024; De Ruysscher, Magerman and Goethals 2024; Chan and Tsui 2023).

Our work contributes a preliminary network understanding of how perceived stigma can influence help-seeking among people with the additional intersecting marginalisation experience of being in contact with the criminal justice system. Higher levels of perceived societal stigma may particularly deter help-seeking among people with fewer, closely connected social contacts, where cultural norms may be tightly monitored and the ‘risk’ of losing people when revealing a stigmatised condition is heightened. This possible deterrent effect of closely interconnected networks has been demonstrated among other populations experiencing social marginalisation (Connell et al., 2024). Longitudinal research is needed to establish whether there is a temporal ordering. i.e., whether societal MH or SU stigma results in people reducing their network size (to protect themselves from uncomfortable interactions), or whether a small network reduces access to perspectives that could challenge stigmatising views and thus perceptions of societal stigma.

Many participants emphasised that if we had spoken to them in the past, their network would have differed in structure and composition, usually by being larger and more populated by others involved in crime and SU**.** Curation of one’s network as a process of re-defining one’s identity is well-documented in SU recovery literature (Francis 2020; Vigdal et al. 2023; Anderson et al. 2021; Dingle et al. 2015), a process which some of our participants had embarked on. In contrast, when living with mental health problems, relationships may gradually wane without this deliberate ‘cutting off’, leaving a core of close-knit support (Perry and Pescosolido 2012). Smaller and closed networks have implications for help-seeking, including a reduction in amount and diversity of knowledge available.

Participants felt that their networks had limited knowledge about what SU problems or MH were, what help existed, and how to access it. Participants with closed networks, particularly small close-knit networks, were at risk of receiving one-sided information that deterred help-seeking or help-seeking from particular places. Among the general population, people are as likely to discuss sensitive topics with close contacts as ‘weak ties’ (Small et al. 2024). Participants in our study often avoided talking about and seeking help for MH or SU help from some contacts who might be considered ‘close’ (e.g., family), but they did not describe approaching people with whom they had less close relationships. Instead participants remained highly selective and instead looked to alternative close contacts (such as a friends or service providers), consistent with other studies about MH help-seeking (Fulginiti et al. 2016, 2022; Perry and Pescosolido 2010, 2015). This may be due to having few peripheral contacts to approach when not involved in employment or wider social settings. Alternatively, compared to less marginalised populations, stigma towards MH and SU may be greater among people in contact with the criminal justice system and their communities, making people wary of help-seeking from less trusted contacts. Supporting people in contact with the justice system to build more diverse networks with multiple components, or engage in activities that increase the chances of making peripheral ties, may assist in introducing them to alternative perspectives and a wider knowledge base.

Network types can be differentiated by the average proportion of network members who were perceived to have supportive or unsupportive attitudes towards help-seeking. We considered average attitudes within a network to reflect network norms, which contribute to planned behaviour (Ajzen 1991). Qualitative findings indicated that participants often shared scepticism with their network members about services (both professional and peer) that deterred help-seeking. This was notable in participants with Entangled and Jaded networks whose history of difficulties and higher levels of shared experiences of MH, SU, and contact with the criminal justice system had led to an accumulation of negative experiences across the network. Distrust of services is well-documented in among people in contact with the criminal justice system (Lafferty et al. 2023) and among individuals with SU and MH challenges, where negative past experiences of services contribute to reluctance to seek help (Lago et al. 2017; Sarah et al. 2015). Enabling people with Entangled and Jaded networks to access more positive, pro-help-seeking cultures is an important consideration, as average appraisal of health providers across network members is associated with MH help-seeking and access (Perry and Pescosolido 2015). It is also vital if encouraging people to seek help, that consideration is given to the impact on relationships with people who oppose help-seeking. People may need support to manage any challenges to their identity and status. Additionally, if a person seeks help, the help-giver must be able to provide a positive experience to avoid further entrenching scepticism and marginalisation.

Levels of MH and SU needs among network members were higher in networks with a greater proportion of friends. Whilst some studies highlight that people with shared experiences are more often approached or considered more approachable, (Perry and Pescosolido 2015; Anderson et al. 2021) this was less clear in our population. Many using peer support espoused its benefits and had transformational experiences. However there were several participants who were sceptical about how helpful others with difficulties would be, had no opportunities to access them, or described harmful experiences which discouraged help-seeking from these types of services (including sabotage of attempts to go substance-free, bullying, rejection when perceived to be struggling, and fear of rejection if their offence was disclosed). Discussion of these harms is limited compared to the positive evaluations of peer support, (Mikolajczak-Degrauwe et al. 2023) potentially reflecting that participants in studies on the topic may be more likely to be those who have had positive experiences. It further highlights the importance of understanding the experience of groups facing additional social marginalisation. Encouraging people in contact with the criminal justice system to approach others with MH or SU needs, or peer support services, therefore needs careful consideration. Whilst this could be potentially life changing, a negative experience may deepen social marginalisation or undermine future help-seeking.

Understanding the reality of the local context in which these networks are embedded is crucial. We found that Jaded networks were commonly in LA1 whilst Building networks were generally in LA2, where the number and range of services was larger. All participants had tried some services, but several had not found one which worked for them. A limited number of services likely reduces peoples’ abilities to find a good-fitting option, and contributes to a position of hopelessness when there appears to be no alternatives. How more rural communities can effectively offer a range of contemporary evidence-based support, and build cultures positive about engaging with them, is a crucial area for consideration and future study.

Interventions to increase early MH and SU help-seeking will likely need to be tailored based on network type, and to consider network and community level needs and awareness. At individual level, our typology can be used by practitioners to reflect on the networks of those they support, ideally together, and hypothesise how the network or specific members within it could best be leveraged to support the person to seek help for MH or SU (or to change other behaviours). It may be useful to inform attempts to add network members who can bring diverse knowledge and attitudes towards help-seeking. Addressing network norms requires network or population level intervention. Population-level interventions, including media-based campaigns, to increase MH and SU awareness, challenge stigma, and promote help-seeking, can impact help-seeking behaviour. However, whether messages reach people experiencing social marginalisation and the differences in effect among groups experiencing marginalisation are underexplored. Our participants reported poor MH and SU knowledge as a barrier to help-seeking. It is therefore vital to develop relatable messaging and credible messengers to ensure communities and networks most in need of support are aware of the support available to them.

### Strengths and limitations

Participants’ comparatively small networks required a bespoke approach to cluster analysis. Our transparently reported mixed-methods design facilitated an exploratory and replicable approach to clustering whereby combining qualitative, quantitative, and network data counterbalanced limitations introduced by using one approach alone, thus increasing the trustworthiness and validity of the typology. Many participants were receiving or seeking support, limiting our ability to capture the contemporary experience of people not receiving or seeking help. However, retrospective exploration of help-seeking when people were not seeking or receiving support services illuminated key experiences. Finally, discussing highly stigmatised conditions among people who experience social marginalisation, particularly where issues of illegal activity are integral, may result in socially desirability bias in participant responses. Some participants were reluctant to name everyone their network, for example when trying to protect associates involved in illegal activity or family reputation. We probed explicitly for network members with whom they may share illegal or social undesirable activities, and achieved this in many cases. However, readers should consider our results to be, at least in part, based on presentations curated by the participants. This is not unique to social network research, but the focus on social networks in this population warrants additional care in research and practice, which we reflect on elsewhere.

### Implications for practice, policy and research

#### Practice

Practitioners may strengthen the impact of interventions to encourage MH and SU help-seeking if they better understand social networks in local contexts, and how they support or constrain help-seeking, and tailor interventions to these relational realities.

Supporting service users with limited social support to build more diverse networks, and access cultures that support identity and status change, may increase successful attempts to seek help for MH and SU problems. This may similarly assist people whose networks are characterised by scepticism and stigma towards help-seeking.

The typology can be utilised to consider network influences on other behaviours that practitioners may seek to change, such as substance use, offending behaviour, or activity participation. Strategies to assist people may differ based on individual circumstances, and how receptive network contacts are for different issues, recognising that help needed might alter if networks changes.

#### Research

Our exploratory approach should be refined with larger samples, and in different contexts.

Longitudinal studies to ascertain the nature and effects of network change on help-seeking and other behaviours would be beneficial.

There is a need to better understand the optimal ways to make a range of evidence-based interventions and supports accessible to communities with different infrastructures and cultures, such as rural contexts or where community stigma towards MH, SU, and help-seeking is high.

### Policy

Public health and other policy responses seeking to achieve network and community-level attitudinal change or public education need to tailor their messages, messengers and delivery methods to make these relatable, credible, and effective for reaching people in communities impacted by high levels of social marginalisation.

## Conclusion

Social network analysis presents a novel method to ascertain a nuanced understanding of the varying social networks of people in contact with the criminal justice system, and their respective influences on behaviour. The social networks of people in contact with the criminal justice system play an important role in transmitting knowledge, attitudes, and norms that support or deter help-seeking for MH and SU. Observing five distinct network types with different impacts on help-seeking suggests various routes for intervention. An appreciation of the structure, composition, and culture of an individual’s social network can inform individual, social network, and community-level interventions. These include creating mechanisms that increase the number of relationships that contribute to a pro-help-seeking network culture and that increase access to diverse knowledge and resources. Greater appreciation of social network influences presents possibilities for intervening at to address attitudinal norms shared by the surrounding network and community that support or deter help-seeking.

## Supplementary Information

Supplementary Material 1

## Data Availability

Data are accessible via the UK Data Service by legitimate researchers with ethical approval and permission from the original study authors. Code is being prepared for sharing on the GitHub platform. Our protocol and related work are available at.https:www.//osf.io/rj53k/ (doi:10.17605/OSF.IO/RJ53K)

## Abbreviations

LA1: Local Authority 1
LA2: Local Authority 2
LEAP: Lived Experience Advisory Panel
M: Mean
MH: Mental health
n: Number
SD: Standard deviation
SU: Substance use
