# Supplementary material for: Help-seeking for mental health and substance use problems among people in contact with the criminal justice system: a mixed-methods social network analysis
Source: Health Justice. 2026 Jun 26;14:42. doi: 10.1186/s40352-026-00431-2 (PMC13595624; doi:10.1186/s40352-026-00431-2)
Supplement: Supplementary file 1 [file 40352_2026_431_MOESM1_ESM.pdf]

Help-seeking for mental health and substance use problems among people in contact with the criminal justice system: a mixed methods social network analysis

Supplementary file

Contents

Coding framework 2
Supplementary Table 1 3
Supplementary Table 2 4

Coding framework

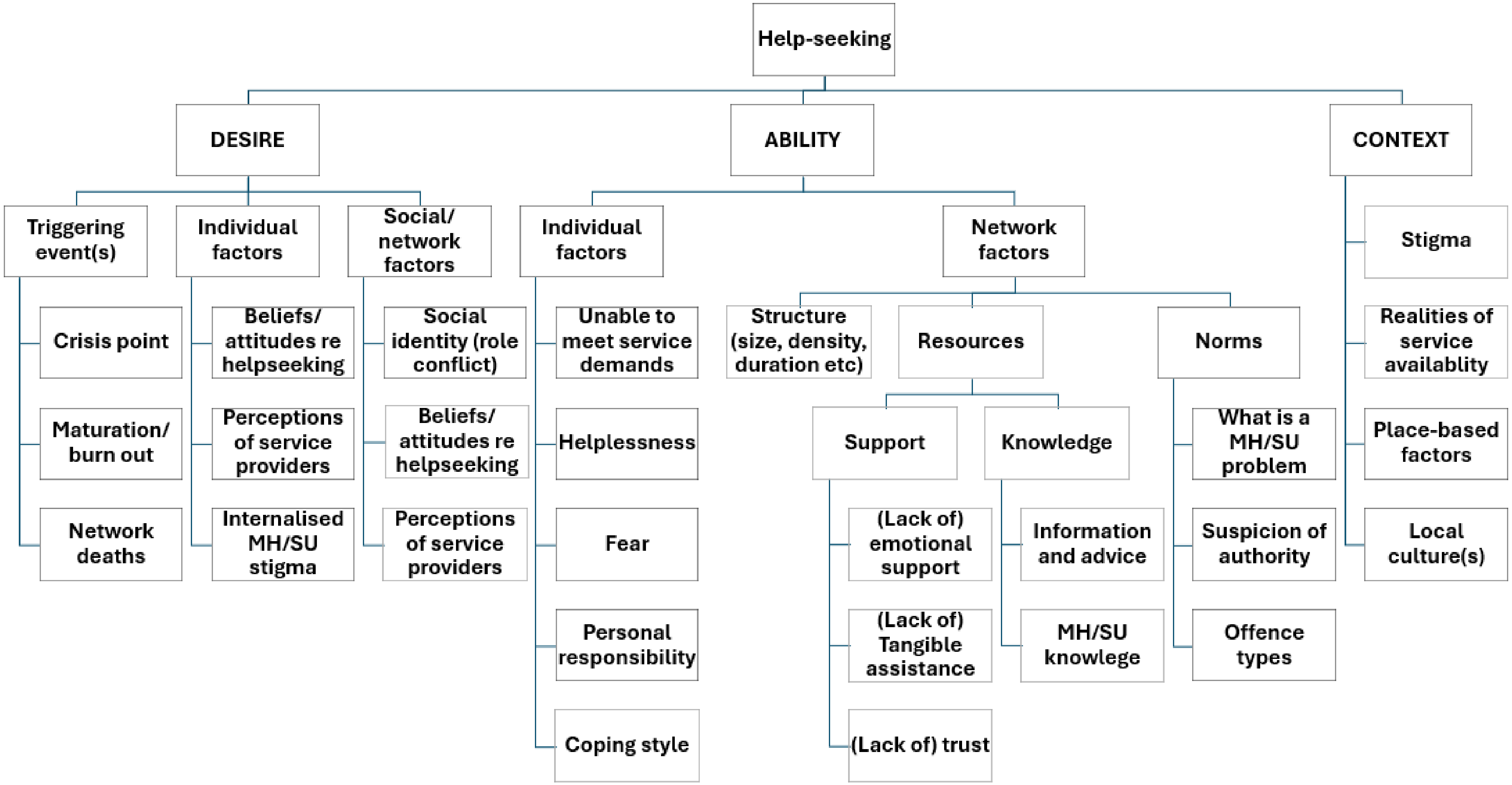

Supplementary Table A. Individual characteristics and measured variables across network types

Characteristic	Entangled N = 10[1]	Close-knit N = 16[1]	Unsure N = 8[1]	Jaded N = 6[1]	Building N = 8[1]
Age	41 (10)	37 (11)	35 (8)	44 (12)	35 (8)
Gender					
Male	80	69	50	83	75
Female	20	31	50	17	25
Ethnicity					
White Scottish	90	94	75	83	88
Other	10	6.3	25	17	13
Local authority					
LA1	50	38	50	83	25
LA2	50	63	50	17	75
Area deprivation level					
20% most deprived	50	88	75	50	100
other	50	13	25	50	0
Has current MH/SU condition	80	100	88	100	88
Has MH/SU support	90	81	50	50	100
Previously had MH/SU support	80	75	75	83	100
Currently help-seeking	40	81	63	33	50
Previous failed help-seeking attempt	80	75	88	100	63
Ever used NHS/LA services	90	69	88	83	63
Ever used peer support	70	44	25	17	75
Type of justice involvement					
Community disposal	70	44	63	83	63
Parole	0	0	0	0	0
Other	30	56	38	17	38
Lifetime community disposals	5 (5)	6 (6)	3 (4)	4 (3)	3 (3)
Lifetime prison sentences	7 (10)	5 (8)	2 (3)	5 (3)	1 (1)
Lifetime justice system contacts without conviction	29 (42)	22 (27)	5 (6)	5 (6)	5 (4)
Current/most recent offence: violence	50	38	50	50	75
Current/most recent offence: sexual	10	13	13	0	0
Current/most recent offence: drug-related	20	31	38	0	50

[1] Mean (SD); %

Supplementary Table B. Individual characteristics and measured variables across network types

Characteristic	Entangled N = 10[1]	Close-knit N = 16[1]	Unsure N = 8[1]	Jaded N = 6[1]	Building N = 8[1]
Network size	15 (3)	9 (4)	6 (1)	12 (3)	6 (2)
Edges	46 (25)	15 (8)	7 (4)	17 (7)	11 (6)
Components					
1	70	63	0	0	75
2	20	13	75	0	25
3	0	25	25	17	0
4	10	0	0	50	0
5	0	0	0	17	0
6	0	0	0	17	0
Edge density (inter-connectedness)	0.41 (0.11)	0.53 (0.23)	0.50 (0.15)	0.25 (0.04)	0.70 (0.26)
Transitivity (within network clustering)	0.67 (0.13)	0.73 (0.12)	1.00 (0.00)	0.90 (0.08)	0.78 (0.21)
Mean path length (compactness)	1.82 (0.34)	1.41 (0.20)	1.00 (0.00)	1.14 (0.13)	1.21 (0.16)
Prop. of network service providers	15 (16)	30 (31)	32 (32)	33 (14)	17 (24)
Prop. of network friends	46 (17)	26 (23)	25 (28)	28 (13)	35 (32)
Prop. of network family	28 (16)	35 (22)	31 (31)	28 (13)	42 (40)
Prop. of network female	39 (21)	47 (17)	57 (21)	44 (13)	55 (31)
Prop. of network known 10+ yrs	52 (11)	50 (29)	42 (26)	50 (22)	46 (37)
Prop. w/ MH experience	67 (25)	56 (27)	48 (17)	43 (28)	65 (34)
Prop. w/ SU experience	49 (29)	46 (29)	31 (30)	40 (23)	59 (32)
Prop. w/ justice-involvement experience	44 (29)	44 (32)	18 (19)	28 (16)	40 (35)
Prop. supportive of MH help-seeking	79 (17)	82 (22)	82 (35)	72 (21)	86 (22)
Prop. unsupportive of MH help-seeking	14 (14)	11 (18)	7 (15)	9 (12)	10 (19)
Prop. supportive of SU help-seeking	78 (17)	81 (17)	94 (12)	76 (21)	90 (19)
Prop. unsupportive of SU help-seeking	15 (17)	8 (12)	6 (12)	12 (12)	10 (19)

[1] Mean (SD); %

Supplementary Table C. Stigma and perceived helpfulness scores overall and across network types

	Overall	Entangled	Close-knit	Unsure	Jaded	Building
n	48	10	16	8	6	8
MH professionals useful (mean (SD))	2.60 (1.33)	2.90 (1.37)	2.56 (1.41)	2.62 (0.92)	2.17 (1.33)	2.62 (1.69)
MH peer support useful (mean (SD))	2.79 (1.09)	3.10 (1.10)	2.50 (1.32)	2.50 (1.07)	2.83 (0.41)	3.25 (0.89)
Friends/family useful for MH support (mean (SD))	2.25 (1.41)	2.40 (1.58)	2.38 (1.36)	2.25 (1.16)	1.83 (1.60)	2.12 (1.64)
Average usefulness of MH support (mean (SD))	2.55 (0.78)	2.80 (0.83)	2.48 (0.92)	2.46 (0.50)	2.28 (0.57)	2.67 (0.85)
SU professionals useful (mean (SD))	2.90 (1.10)	3.30 (1.06)	2.50 (1.15)	2.88 (0.35)	2.50 (1.38)	3.50 (1.07)
SU peer support useful (mean (SD))	2.79 (1.18)	3.10 (1.20)	2.50 (1.37)	2.50 (1.07)	2.83 (0.98)	3.25 (1.04)
Friends/family useful for SU support (mean (SD))	2.21 (1.44)	2.60 (1.51)	2.19 (1.47)	2.38 (1.30)	1.33 (1.51)	2.25 (1.49)
Average usefulness of SU support (mean (SD))	2.63 (0.88)	3.00 (0.83)	2.40 (1.10)	2.58 (0.46)	2.22 (0.83)	3.00 (0.62)
Perception MH stigma (mean (SD))	1.49 (0.45)	1.37 (0.35)	1.74 (0.42)	1.47 (0.42)	1.47 (0.40)	1.14 (0.51)
Perception SU stigma (mean (SD))	1.86 (0.36)	1.74 (0.36)	1.99 (0.41)	1.92 (0.38)	1.69 (0.15)	1.78 (0.27)

Data represents mean (SD)
